# Prosodic Entrainment in Conversations of Verbal Children and Teens on the Autism Spectrum

**DOI:** 10.3389/fpsyg.2020.582221

**Published:** 2020-10-08

**Authors:** Heike Lehnert-LeHouillier, Susana Terrazas, Steven Sandoval

**Affiliations:** ^1^Department of Communication Disorders, New Mexico State University, Las Cruces, NM, United States; ^2^Klipsch School of Electrical and Computer Engineering, New Mexico State University, Las Cruces, NM, United States

**Keywords:** autism, prosody, conversational speech, children, adolescents

## Abstract

Unusual speech prosody has long been recognized as a characteristic feature of the speech of individuals diagnosed with Autism Spectrum Disorders (ASD). However, research to determine the exact nature of this difference in speech prosody is still ongoing. Many individuals with verbal autism perform well on tasks testing speech prosody. Nonetheless, their expressive prosody is judged to be unusual by others. We propose that one aspect of this perceived difference in speech prosody in individuals with ASD may be due to a deficit in the ability to entrain—or become more similar—to their conversation partners in prosodic features over the course of a conversation. In order to investigate this hypothesis, 24 children and teens between the ages of 9 and 15 years participated in our study. Twelve of the participants had previously been diagnosed with ASD and the other 12 participants were matched to the ASD participants in age, gender, and non-verbal IQ scores. All participants completed a goal-directed conversation task, which was subsequently analyzed acoustically. Our results suggest (1) that youth diagnosed with ASD entrain less to their conversation partners compared to their neurotypical peers—in fact, children and teens diagnosed with ASD tend to dis-entrain from their conversation partners while their neurotypical peers tend to converge to their conversation partners’ prosodic features. (2) Although age interacts differently with prosodic entrainment in youth with and without ASD, this difference is attributable to the entrainment behavior of the conversation partners rather than to those with ASD. (3) Better language skill is negatively correlated with prosodic entrainment for both youth with and without ASD. The observed differences in prosodic entrainment in children and teens with ASD may not only contribute to the perceived unusual prosody in youth with ASD but are also likely to be indicative of their difficulties in social communication, which constitutes a core challenge for individuals with ASD.

## Introduction

Autism Spectrum Disorder (ASD) refers to a spectrum of neuro-developmental disorders characterized by persistent deficits in social communication and interaction as well as the presentation of restricted and repetitive patterns of behavior, interests, or activities (American Psychiatric Association, 2013). While some individuals diagnosed with ASD do not develop spoken language, approximately 70% of children with ASD acquire some spoken language, and close to half of children on the autism spectrum attain fluent speech (Wodka et al., 2013). The observation that those with ASD, who are able to speak, often seem to have unusual prosody, dates back to the earliest descriptions of the disorder (i.e., Kanner, 1943; Asperger, 1944). However, the exact nature of the differences in speech prosody in those with verbal autism is as of yet not completely understood. In particular the link between speech prosody deficits and the difficulties in social communication in those diagnosed with the disorder remains an area in need of further investigation.

Most generally, speech prosody encompasses changes in speech characteristics such as fundamental frequency (f0), timing, and amplitude to convey grammatical, pragmatic, emotional, and conversational meaning. Prosodic categories that are used to convey grammatical meaning include ***stress*** which, for example, can alter the meaning from noun (i.e., PROduce) to verb (i.e., proDUCE) at the word level, and ***intonation*** which can help, for example, to distinguish prosodically between statements and questions (i.e., You saw Mary. vs. You saw Mary?). Statements are frequently marked by a falling intonation whereas questions, especially yes/no questions, tend to be expressed with a rising intonation. Both stress and intonation can also be means to express pragmatic meaning. For example, changes in sentence stress or accent (i.e., “You saw MARY?” vs. “YOU saw Mary?”) have a contrastive function and are governed by pragmatic context. The first question with the sentence stress on “Mary” is most felicitous in a situation where the information that “Mary” was seen comes as a surprise. The second example with the sentence stress on “You” is most appropriate in a situation, in which it is doubtful that the speaker saw Mary. Emotional or affective prosody is also sometimes expressed via intonation patterns, but is more commonly associated with global prosodic characteristics (cf. Banse and Scherer, 1996). Finally, the use and function of prosody in conversation is associated with turn-taking events as well as continuation or changes in the topic of the conversation, but also encompasses the expression of attitudes of the conversation partners toward the topic or each other (Ward, 2019). Furthermore, conversational prosody functions as a means to modulate social interactions via aligning/entraining to prosodic features of a conversation partner (i.e., Gregory et al., 1997; Levitan et al., 2012; Lubold and Pon-Barry, 2014). However, regardless of whether prosody serves a grammatical, pragmatic, emotional, or conversational function, the predominant—although not the only—acoustic marker of prosody in spoken language is fundamental frequency (f0), and its perceptual correlate, pitch, serves as one of the major perceptual cues in prosody perception. Given that the speech of individuals with ASD has been characterized as “robotic” and “monotone” in many earlier descriptions of language in those with ASD (i.e., Fay and Schuler, 1980; Frith, 1991), studies of both production and perception of speech prosody have set out to understand this atypical feature of the speech of individuals on the autism spectrum.

The focus of the research on the perception and production of prosody in those with verbal autism has thus far focused on grammatical (Simmons and Baltaxe, 1975; Shriberg et al., 2001; Paul et al., 2005a; Filipe et al., 2014; Diehl et al., 2015; among others), pragmatic (Baltaxe, 1984; Paul et al., 2005b; Peppé et al., 2006; DePape et al., 2012; among others), and emotional/affective prosody (Rutherford et al., 2002; Grossman and Tager-Flusberg, 2012; Kjelgaard and Tager-Flusberg, 2013; Hubbard et al., 2017; among others). What is striking when considering the results of previous studies is the heterogeneity of the reported findings. Although McCann et al. (2007) report that all their participants with ASD showed prosodic deficits in at least one of the three prosodic areas mentioned above, there is much evidence to suggest that only a sub-set of those with ASD exhibit prosodic differences. For example, Simmons and Baltaxe (1975) report that only 4 of their 7 participants showed differences in prosody, and Paul et al. (2005b) found that the difference between their participants with ASD and the neurotypical control group did not rise to the level of statistical significance, although the two groups did differ significantly in terms of the production and perception of stress patterns. A difference in the production of stress was also reported by Shriberg et al. (2001) who found that between approximately 26 and 54% of their participants with ASD were rated as exhibiting unusual stress patterns compared to only about 6% of the control group. Differences in prosody in those with and without ASD are also present when looking at more global measures of prosody, such as mean f0 and f0 range over longer discourse such as narratives. For example, Diehl et al. (2009) report that there was no difference in mean f0 between the participants with ASD and the control group during narratives, but the participants on the autism spectrum showed a greater f0 range. However, a more recent study looking at narratives found no differences in either mean f0 nor f0 range between those with and without ASD (Patel et al., 2020). However, Patel et al. (2020) do report a marginally significant difference in utterance final f0 excursion between those with and without ASD. Contradictory results like these are not uncommon – although differences in grammatical, pragmatic, and emotional prosody at word, sentence, and conversational level have been reported, some of the findings of earlier studies have not been replicated. For example, while Fosnot and Jun (1999) found atypical intonation pattern in their participants with ASD, Baltaxe et al. (1984) do not report atypical intonation in the speech of their participants on the autism spectrum. Similarly, Grossman et al. (2010) report that their participants with ASD did not differ in the perception of affective prosody compared to their typically developing peers whereas Diehl and Paul (2013) report a difference in the perception of affective prosody between their participants with and without ASD. Some of this observed heterogeneity may be due to methodological differences between the studies such as age of participants, sample size, absence of normative data, as well as the use of subjective as opposed to objective measures (see Paul et al., 2005b for a short discussion). However, the contradictory findings of studies on prosody in those with ASD may also be attributable to the heterogeneity of the population itself and point to a need to look deeper to uncover correlations with other traits, in addition to an ASD diagnosis, that may predict differences in the production and perception of prosody. For example, Green and Tobin (2009) identify three sub-groups in their participants with ASD—those who showed a typical pitch range, those who exhibited a narrower than normal pitch range, and those who had a wider than typical pitch range. DePape et al. (2012) were able to tie the differences between narrow pitch range use in those with ASD to moderate overall language performance, while ASD participants exhibiting wider pitch ranges were found to show overall stronger language skills. Filipe et al. (2018) were able to show that prosodic deficits were associated with executive function deficits, especially divided attention, working memory, set-switching, and inhibition. Interestingly, this association between executive function and prosodic performance was found for both the participants with and without ASD. However, the participants on the autism spectrum scored overall lower on both prosody tasks and the earlier mentioned executive function tasks.

In addition to the need to investigate cognitive and developmental underpinnings of atypical prosody to explain the heterogenic patterns of prosody production and perception in the speech of individuals with ASD, there is also a need to look at speech prosody in ASD from a conversational perspective. While it is true that incorrect lexical stress placement or atypical use of prosody to mark information structure may prevent those with ASD to get their intended message across effectively, and possibly confuse their conversation partners, this in itself does not seem quite sufficient to prevent those with ASD from developing meaningful friendships or from successfully completing a job interview. This becomes especially evident when considering that even those with ASD, who do not show prosodic deficits on tests of prosody, are still perceived as having unusual prosody (Nadig and Shaw, 2012; Filipe et al., 2014). Therefore, we investigate the production of conversational prosody with particular emphasis on the relational function of prosody at the conversational level in the current study.

Among the studies of prosody in individuals with ASD, several have investigated aspects of conversational prosody. Nadig and Shaw (2012) included two tasks to elicit conversational speech; one during which and adult research assistant asked the study participants questions, and during the second task participants had to instruct the adult research assistant to pick an object out of a set of four by describing the object. Sharda et al. (2010) asked the participants in their study open ended questions about preferences and likes; i.e., “Do you like ice cream?” in order to elicit the participants speech. Green and Tobin (2009) also used open ended questions to elicit responses from their participants. All three studies on conversational prosody in individuals with ASD have investigated mean f0 and f0 range and found greater f0 range and variability of f0 in individuals with ASD compared to neurotypical controls. However, all of these studies looked at the different f0 measures of the participants with ASD only—without taking speaker characteristics of the conversation partners into account. Furthermore, only a subset of selected utterances of the speakers with ASD (or the control group speakers) were used for acoustic analysis, rather than the majority of the conversational speech. However, in addition to the grammatical, pragmatic, and affective function of prosody discussed above, speech prosody is also used to modulate human interactions in conversation (see Beňuš, 2014; Levitan et al., 2015; Ward, 2019). Hence, in order to investigate the relational aspect of conversational prosody, it is important to analyze the speech of all conversation partners. The research presented here investigates one well-known prosodic characteristic of conversational speech that serves to mediate the relationship of the conversation partners, namely prosodic entrainment/alignment over the course of a conversation.

Conversational entrainment or alignment, in general, refers to the process during which speakers attune to each other via aligning their linguistic style during communicative interactions. Entrainment can be realized as either a continuous increase in proximity over the course of the conversation such that conversation partners are more similar in a given linguistic feature later in the conversation when compared to the beginning of the conversation, a process referred to as convergence, or it can be an ongoing process of the coordination of linguistic features during which conversation partners vary linguistic features in a parallel manner—often referred to as synchrony (Levitan and Hirschberg, 2011). Convergence can happen either linearly over the course of an entire conversation (i.e., Gregory et al., 1997; Collins, 1998; Kousidis et al., 2009; Levitan and Hirschberg, 2011), or at the level of the conversational turn (i.e., Levitan and Hirschberg, 2011; Reichel et al., 2018; Weise and Levitan, 2018). Hence, accommodation has been shown to increase linearly over the course of an entire conversation as well as to vary from turn to turn. For the purpose of the current study, we focus on linear convergence in prosodic features over the course of a conversation only.

Conversational entrainment behavior forms the empirical basis for theoretical models, such as the Communication Accommodation Theory (CAT) (Giles et al., 1991) or other models of dialogue behavior (Pickering and Garrod, 2004), that try to capture whether and how speakers modulate social and relational distance in conversations. Within such theories of the dynamics of interpersonal communication, the convergence or entrainment of speakers in linguistic characteristics during conversational interactions is typically equated with a variety of positive attributes regarding the conversation itself as well as the participating speakers’ identity. These positive attributes range from increased effectiveness and mutual understanding to an increase in perceived attractiveness of the speakers. On the other hand, divergence or dis-entrainment in linguistic style as well as maintenance—the lack of accommodation of the linguistic behaviors of a conversation partner—are typically associated with negative relational attributes. For an extensive, more recent review of CAT and its interdisciplinary applications (see Soliz and Giles, 2014). Speakers have been shown to exhibit a variety of accommodative linguistic behaviors. For example, speakers may align at the lexical level by entraining in terms of word choice (i.e., Brennan and Clark, 1996; Nenkova et al., 2008), at the syntactic level by becoming more similar in the use of syntactic constructions over the course of conversations (i.e., Cleland and Pickering, 2003; Branigan et al., 2007), and at the phonetic level via increased similarity in phonetic-acoustic features (i.e., Pardo, 2006; Pardo et al., 2017; Lewandowski and Jilka, 2019). Conversational entrainment has also been observed in a variety of acoustic measures related to speech prosody; speakers entrain, for example, in speaking rate (Local, 2007; Levitan et al., 2012), intensity (Natale, 1975; Levitan et al., 2012), pause duration (Edlund et al., 2009), and fundamental frequency (Gregory and Webster, 1996; Gregory et al., 1997; Levitan and Hirschberg, 2011; Babel and Bulatov, 2012; among others). Similar to other linguistic accommodation behaviors, prosodic entrainment has been shown to correlate with a variety of positive attributes regarding the conversation itself as well as the participating speakers. Prosodic entrainment, especially f0 entrainment, has shown to correlate with the perceived quality of a conversation (Michalsky et al., 2018), the rapport between conversation partners (Lubold and Pon-Barry, 2014), perceived attractiveness and likability (Michalsky and Schoormann, 2017), as well as the ability to engage successfully in teamwork (Niebuhr and Michalsky, 2019).

Given that impairment in social and relational communicative abilities is one of the core characteristics of ASD (American Psychiatric Association, 2013), the question arises whether those with ASD differ from neurotypical individuals in conversational prosodic features. Entrainment behaviors in those with ASD have been investigated with respect to lexical entrainment and entrainment in speaking rate. The results of studies on lexical entrainment all concur that individuals with ASD show spontaneous lexical entrainment comparable to neurotypical communicators (Nadig et al., 2015; Branigan et al., 2016; Hopkins et al., 2017). However, Nadig et al. (2015) report that while the participants with ASD were just as efficient as the controls, they were slightly slower and were less likely to incorporate the conversation partners’ contributions into their descriptions. Looking at prosodic entrainment behavior, Wynn et al. (2018) investigated speaking rate in individuals with ASD—both children and adults—and found that only neurotypical adults exhibited entrainment in speech rate in their study. Wynn et al.’s (2018) study was an important first step in the investigation of prosodic entrainment behaviors in those with ASD. The study examined speech rate entrainment using a quasi-conversational design where participants watched a pre-recorded female talker on a computer screen who asked them to describe what they saw in a picture. The participants then described the picture, and these descriptions were analyzed for speech rate entrainment to the female speaker, who had instructed them. This experimental set-up does not lend itself to draw conclusions about the relational aspect of the entrainment behavior as it somewhat lacks in ecological validity (see Pardo (2006) for a discussion of entrainment/convergence in shadowing as opposed to conversational tasks). Therefore, prosodic entrainment in individuals with ASD, especially as it relates to conversational and interpersonal aspects, needs further investigation.

The current study set out to investigate prosodic entrainment in children and teens with and without ASD by investigating mean fundamental frequency (f0) and f0 range convergence over the course of a conversation. We chose to look at f0 because, as noted above, f0 has been shown to correlate with both quality of the conversation as well as personal and interpersonal attributes of the conversation partners. Therefore, entrainment (the convergence of speakers in f0), dis-entrainment (the divergence of speakers in f0), or maintenance (no change in f0) have the potential to signal to conversation partners information about a speaker that may lead them to view the speaker in a positive or negative light, and possibly lead to positive or negative interpersonal outcomes (see Ireland et al., 2011). For the purpose of investigating f0 entrainment, we collected conversational data from children and teens diagnosed with ASD and neurotypical peers matched on age, gender, and non-verbal IQ scores. We extracted mean f0 and f0 range measures with the following goals: (1) To compare mean f0 and f0 range entrainment in youth with and without ASD—both for the conversation as well as for each individual conversation partner; (2) to investigate whether individual differences in entrainment behavior are related to other variables such as overall language ability, age, or non-verbal IQ. We include the variables of language ability and non-verbal IQ in addition to investigating whether prosodic entrainment varies between the two groups because prior studies have shown that these speaker characteristics may contribute to variability in speech prosody in those with ASD (i.e., DePape et al., 2012; Nadig and Shaw, 2012). We also include age as a variable because some studies have reported that entrainment decreases with age in children and adolescents (i.e., Nilsenova et al., 2009).

Based on the research on prosody in individuals with ASD and the core deficits in social communication in this population, we predict that children and adolescents with ASD will show less f0 entrainment compared to their neurotypical peers. We expect that the relationship between prosodic entrainment and other speaker characteristics such as language ability, non-verbal IQ, and age will be similar to those found between these variables and other prosodic characteristics investigated in prior studies, as described above.

## Materials and Methods

### Participants

Twenty-four children and teens between 9 and 15 years of age participated in the current study. Twelve of the participants (3 girls, 9 boys) carried a formal diagnosis of ASD (Autism = 6, High Functioning Autism = 4, Asperger’s Syndrome = 1, PDD-NOS = 1), and the other 12 participants were neurotypical peers matched to the ASD participants in age, gender, and non-verbal IQ (see Table 1). Participants for this study were recruited from the Las Cruces, Southern New Mexico area, which is a linguistically and culturally diverse region. However, all participants included in this study came from households where English was the first and primary language. All participants passed hearing screenings at pure-tone frequencies of 500, 1,000, 2,000, and 4,000 Hz, and reported normal vision. Participants were administered the Core Language sub-test of the Clinical Evaluation of Language Fundamentals, Fifth Edition (CELF-5; Wiig et al., 2013) to assess general language functioning, as well as the Kaufman Brief Intelligence Test (KBIT-2; Kaufman and Kaufman, 2004) to determine non-verbal and composite IQ scores. The participants on the autism spectrum were furthermore administered the Autism Diagnostic Observation Schedule, Second Edition (ADOS^TM^-2; Lord et al., 2012) to verify the ASD diagnosis. The first author, who is a licensed and certified speech-language pathologist and who was also trained in the administration of the ADOS^TM^-2, administered the standardized tests. A summary of participant characteristics is shown in Table 1.

**TABLE 1:** Summary of group characteristics for the ASD and NT participants. KBIT-2 and CELF-5 scores provided as standard scores.

	ASD group (*n* = 12)		Neurotypical group (*n* = 12)		*p-*value
	Mean (*SD*)	Range	Mean (*SD*)	Range	
Age	12.14 (1.84)	9.01–14.05	12.23 (1.89)	9.01–15.00	>0.05
Non-verbal IQ (KBIT-2)	107 (9.8)	95–128	110 (9.05)	88–120	>0.05
Composite IQ (KBIT-2)	99 (16.76)	76–138	112 (12.57)	87–131	<0.05
Language (CELF-5)	88 (12.43)	73–113	109 (11.56)	89–126	<0.001
ADOS-2	9 (3.41)	7–19	N/A	N/A	N/A

Only participants with average non-verbal IQ scores of 85 or higher were included in the study to guarantee normal cognitive functioning in the non-verbal domain. Furthermore, participants with a composite IQ score of 75 or lower were excluded from the study to assure that participants were able to successfully engage in the study tasks. The two groups were matched on age (ASD: *M* = 12.14, *SD* = 1.84; NT: *M* = 12.23, *SD* = 1.89), gender (3 female and 9 male participants per group), and non-verbal IQ (ASD: *M* = 107, *SD* = 9.8; NT: *M* = 110, *SD* = 9.05), but differed significantly in composite IQ scores [*F*(1, 22) = 5.11, *p* = 0.03, *d* = 0.92] and language functioning [*F*(1, 22) = 18.08, *p* < 0.001, *d* = −1.74].

### Materials and Procedure

The research reported in this study was approved by the New Mexico State University Institutional Review Board and conforms with the guidelines of the Office of Research Integrity and Ethics. The legal guardians of the participants provided written informed consent and the participants themselves provided written assent to participate in this study. After administration of the hearing screening and the above listed standardized tests (CELF-5, KBIT-2, and ADOS-2—if applicable), each participant was seated in a sound-treated room where a goal-oriented conversation between the participant and one of nine undergraduate assistants was recorded. In order to elicit the goal-oriented conversation, the participants were asked to complete the Diapix task (Baker and Hazan, 2011), during which each conversation partner is given a picture which is similar but not identical to the conversation partner’s picture. The conversational pair was then asked to find the differences between their respective pictures through collaborative conversation without being allowed to see each other’s pictures.

The conversations were recorded in audio wave file format at a 44.1 kHz sampling rate with 16-bit resolution using a Marantz PMD 670 digital recorder and a Shure SM58 cardioid dynamic microphone that was placed between the conversation partners, approximately 30 inches from each speaker. The audio files were then transferred onto desktop computers for post-processing and labeling. Each sound file was annotated by hand using Praat (Boersma and Weeninck, 2019). TextGrid files to label all utterances produced by each speaker during the conversation, rather than employing an automated signal extraction procedure to ensure that f0 measures were extracted only from verified speech samples. This was necessary since some individuals with ASD will occasionally produce non-speech sounds—a form of vocal stereotyped behaviors—which are difficult to recognize when automatically extracting interpausal units. As a result of the annotation by hand, only those utterances that were verified as linguistically meaningful were included in the analysis. Hence, all non-linguistic vocalizations such as laughter, humming, vocal stereotypy, etc. as well as silences were excluded from the subsequent acoustic analysis during which measures of mean f0 and f0 range were extracted from the first and the last third of each conversation for each of the speakers in the conversational dyad.

### Acoustic Analysis and Entrainment Measures

As noted above, rather than looking at entrainment at the level of conversational turns, this study investigates prosodic entrainment as progressive decrease in distance of f0 measures to assess convergence by comparing earlier segments to segments later in the conversation. Due to well established differences in terms of topic maintenance during conversations as well processing differences that frequently lead to delayed responses in those with ASD, we decided against a turn-level entrainment analysis using interpausal units for the current study, as these characteristics may have rendered such an analysis unreliable.

In order to obtain early and late measurements of mean f0 and f0 range from each of the speakers, conversations were divided into thirds. Using the initial and final third of a conversation to extract measurements of phonetic features for the purpose of assessing convergence in these features is one of the methods used in prior studies (Kim et al., 2011). For the first and last third of each conversation, the fundamental frequency was automatically estimated every 10 ms using Praat (Boersma and Weeninck, 2019) from all utterances produced by each speaker during these thirds. The f0 range (pitch floor and pitch ceiling) used for the analysis was adjusted for each speaker class (children, teenage girls, and teenage boys). Specifically, we used f0 ranges of 100–450 Hz for children (Pedersen et al., 2015), of 100–400 Hz for teenage girls (Sorenson, 1989), and of 50–350 Hz for teenage boys (Pedersen et al., 2015). Then, using the f0 values extracted over the first and last third of the conversion, two features (*k* = 1,2) were computed for each speaker. Each feature is parameterized by a vector drawn from the start point (corresponding to the computed value for the initial third) $S^{\left(k\right)}=\left(S1_{s}^{\left(k\right)},S2_{s}^{\left(k\right)}\right)$ to the end point (corresponding to the computed value of the last third) $E^{\left(k\right)}=\left(S1_{e}^{\left(k\right)},S2_{e}^{\left(k\right)}\right)$ where S1 and S2 indicate speaker one vs. speaker two of each conversational dyad; the subscripts *s* and *e* indicate the first vs. the last third; and *k* indicates the feature type. The first feature (*k* = 1) measures the change in mean fundamental frequency and the second feature (*k* = 2) measures the change in interquartile range of the fundamental frequency from the first and the last third of the conversation. Next, we defined two lines *l*_*1*_ and *l*_*2*_ corresponding to matching fundamental frequencies *S*2^(*k*)^ = *S*1^(*k*)^ and “half matching” fundamental frequencies $S2^{\left(k\right)}=\frac{1}{2}S1^{\left(k\right)}$. The line *l*_*2*_ is used for adult female conversation partners and teenage boys. In order to determine whether the speech features have become more (or less) similar to each other, we computed both the minimum distance from the point *S*^(*k*)^ to either *l*_*1*_ or *l*_*2*_, and the minimum distance from the point *E*^(*k*)^ to either *l*_*1*_ or *l*_*2*_. We denote the minimum distance from *S*^(*k*)^ to the line as $d_{1}^{\left(k\right)}$ and the minimum distance from *E*^(*k*)^ to the line as $d_{2}^{\left(k\right)}$ The difference in minimum distance between the first and last thirds of the conversation are used as measure of change in entrainment,

$$\Delta ent=d_{1}^{\left(k\right)}-d_{2}^{\left(k\right)}.$$

Finally, in order to assess the contribution of each speaker to the change in entrainment, we assign a percentage which we define as a responsibility measure for each speaker. Each speaker’s responsibility for the change in the *k*th feature is given by:

$$S1_{resp}^{\left(k\right)}=\frac{d_{3}^{\left(k\right)}}{d_{3}^{\left(k\right)}+d_{4}^{\left(k\right)}}$$

and

$$S2_{resp}^{\left(k\right)}=\frac{d_{4}^{\left(k\right)}}{d_{3}^{\left(k\right)}+d_{4}^{\left(k\right)}}$$

where

$$d_{3}^{\left(k\right)}=\left|S1_{e}^{\left(k\right)}-S1_{s}^{\left(k\right)}\right|$$

and

$$d_{4}^{\left(k\right)}=\left|S2_{e}^{\left(k\right)}-S2_{s}^{\left(k\right)}\right|.$$

**FIGURE 1:**
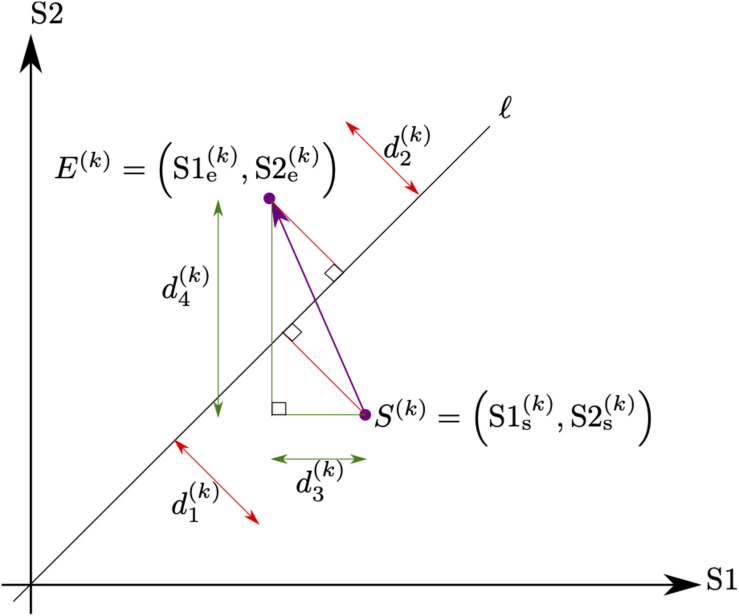
Illustration of the geometry associated with the proposed change in entrainment measurement. Each feature (k = 1,2) is parameterized by the (purple) vector drawn from the start point *S^(k)^* = *(S1${}_{s}^{\left(k\right)}$,S2${}_{s}^{\left(k\right)}$)* which represents the value of both speaker’s f0 measurement over the first third of the conversation to the end point E*^(k)^* = *(S1${}_{e}^{\left(k\right)}$,S2${}_{e}^{\left(k\right)}$)*, which represents the value of both speaker’s f0 measurement over the last third of the conversation. The difference between the minimum distance from the point *S*^(k)^ to the line denoted as d*${}_{1}^{\left(k\right)}$* and the minimum distance from the point *E*^(k)^ to the line denoted as *d${}_{2}^{\left(k\right)}$* gives the change in entrainment measurement. Finally, the individual speaker contribution to the change in entrainment is assessed by computing the ratio of *d${}_{3}^{\left(k\right)}$* = *|S1${}_{e}^{\left(k\right)}$–S1${}_{s}^{\left(k\right)}$*| and *d${}_{3}^{\left(k\right)}$* = |*S1${}_{e}^{\left(k\right)}$–S1${}_{s}^{\left(k\right)}$*| to the sum *d${}_{3}^{\left(k\right)}$* + *d${}_{4}^{\left(k\right)}$*, which represents the proportion of the change along the *x*-axis (Speaker 1) and *y*-axis (Speaker *2)*, respectively.

The process of obtaining the described entrainment measures and relationship between the individual measures are illustrated in Figure 1.

### Statistical Analysis

In order to assess the differences in conversational prosodic entrainment between youth with and without ASD as well as relationships between entrainment and overall language ability, age and non-verbal IQ of the participants, multiple linear regressions were carried out. Linear regression models were created to explore group differences in terms of (1) overall prosodic entrainment over the course of the conversation, in terms of (2) the contribution of each individual participant to conversational entrainment (or dis-entrainment), in terms of (3) each participant’s entrainment contribution adjusted for overall conversational entrainment, and (4) the relationships between the three different entrainment measures and language ability, age and non-verbal IQ scores. We did not expect gender differences seen that our participants were children, and we also did not expect that which research assistant the participant talked to would impact the entrainment behavior of our participant. Nonetheless, we included participant gender and conversation partners in the regression models to confirm that that neither of these variables affected the results. If the conversational dyad entrained to each other, the value for conversational entrainment yielded a positive number, if, on the other hand, conversation partners dis-entrained—or became less similar—the conversational entrainment value was negative, and values close to zero (1 Hz or less) indicate no entrainment (i.e., maintenance).

As mentioned in the previous section, in addition to overall conversational f0 entrainment measures, two variables for measures of each individual’s entrainment contribution were created: one for entrainment contribution—which corresponds to the percentage of entrainment (positive value) or dis-entrainment (negative value) that each participant contributed to the overall conversational entrainment for each conversation. Assessing individual entrainment contribution is necessary since it is possible that two conversation partners become more similar over the course of the conversation—i.e., entrainment occurs—but only one of the conversation partners contributes to this entrainment. In other words, it is conceivable that one of the speakers only changes slightly while the other conversation partner does all the work and contributes most of the change. Hence, the entrainment contribution measure is an individualized measure that captures the amount that each participant contributes to the convergence in prosodic features. On the other hand, if the conversational dyad only showed a relatively insignificant amount of overall conversational entrainment during the conversation, but one participant contributed greatly to this relatively small convergence, the entrainment contribution variable may—in this case falsely—attribute a large degree of entrainment to this speaker. In order to adjust for the effect of magnitude of the overall degree of conversational entrainment, we created the adjusted entrainment contribution variable by multiplying the amount of overall conversational entrainment by each speaker’s individual entrainment contribution.

Different linear regression models were then created to explore the entrainment in mean f0 and the entrainment in f0 range. All statistical analyses were conducted in R (R Core Team, 2016) using the packages “tidyverse” (Wickham et al., 2019), “car” (Fox and Weisberg, 2019), “effsize” (Torchiano, 2020), and “lme4” (Bates et al., 2015). The models reported below met assumptions of homogeneity of variance and linearity, and visual inspection of residual plots showed no obvious violations of the assumption that the data were normally distributed.

## Results

Since several of the previous studies on speech prosody in those with ASD assessed mean fundamental frequency and fundamental frequency range (i.e., Diehl et al., 2009; Nadig and Shaw, 2012; Patel et al., 2020), we compared both groups with respect to these measures. No significant group difference in mean f0 was observed [*F*(1, 22) = 2.58, *p* = 0.12]. However, we did observe a significant difference of f0 range between the two groups [*F*(1, 22) = 4.69, *p* = 0.042, *d* = 0.88]. Next, entrainment results are discussed first for mean f0 and then for f0 range.

### Mean Fundamental Frequency Entrainment

#### Mean f0 Conversational Entrainment

**FIGURE 2:**
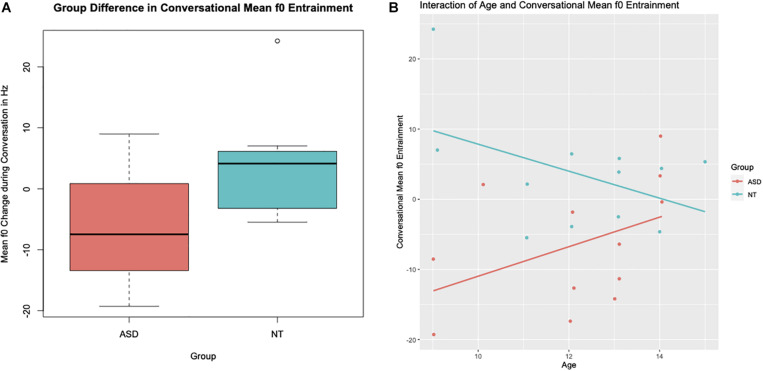
**(A)** Group differences between children and teens with Autism Spectrum Disorder (ASD) and their neurotypical peers (NT) in mean f0 entrainment. **(B)** Relationship between age in years and mean f0 entrainment and its group interaction.

Looking at overall conversational entrainment in mean f0, we found that conversation partners converged in f0, showing more than a 2 Hz convergence, over the course of the conversation in three (25%) out of the 12 conversations with ASD participants, compared to 8 (66%) conversations in the group of neurotypical peers. A significant relationship between group and mean f0 entrainment was observed (*b* = 66.81, *SE* = 21.4, *t* = 3.12, *p* < 0.01). While the conversations involving participants with ASD showed on average 6 Hz dis-entrainment, neurotypical participants and their conversation partners converged on average by 4 Hz—yielding a total 10 Hz difference in conversational entrainment between the groups. The group difference is shown in Figure 2A below. A significant interaction between “Group” (ASD vs. NT) and “Age” was also found (*b* = −4.22, *SE* = 1.71, −2.46, *p* = 0.02). While older participants with ASD entrained more in mean f0 compared to younger participants with ASD, older neurotypical participants tended to entrain less than younger neurotypical participants. The interaction between “Group” and “Age” in conversational mean f0 entrainment is shown in Figure 2B.

Age of the participants (*b* = 2.13, *SE* = 1.23, *t* = 1.73, *p* = 0.09) and CELF Core Language scores (*b* = −0.26, *SE* = 0.13, *p* = 0.06) were approaching significance, and, therefore, were kept in the regression model. However, no significant relationships were found between mean f0 entrainment over the course of the conversation and gender of the participant, non-verbal IQ, or conversation partner. The linear regression model containing the variables “Group” (ASD vs. NT), “Age,” “CELF,” and the interaction of “Age”^∗^“Group” explains 52% (Adjusted *R*^2^ = 0.517) of the variance in mean f0 entrainment over the course of the conversation found in our data set. Group differences alone describe 28% (Adjusted *R*^2^ = 0.276) of the variance in mean f0 entrainment.

#### Mean f0 Entrainment Contribution

**FIGURE 3:**
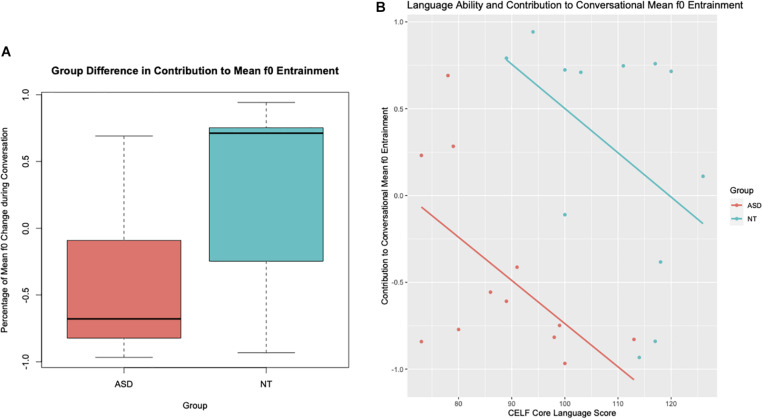
**(A)** Group Differences between children and teens with Autism Spectrum Disorder (ASD) and their neurotypical peers (NT) in the contribution to mean f0 entrapment provided in percent of total entrainment **(B)** Relationship between language ability as measured by the Core Language sub-test of the CELF and contribution to mean f0 entrainment.

In terms of how much each participant contributed to the mean f0 entrainment during the conversation, we found again a significant relationship between mean f0 entrainment contribution and group (*b* = 1.24, *SE* = 0.3, *t* = 4.11, *p* < 0.001). As described above, we measured entrainment contribution as percentage of total change in f0 over the conversation for which the speaker was responsible. If, for example, both speakers converged by 10 Hz over the course of the conversation, and one of the participant’s f0 changed 4 Hz, this participant’s entrainment contribution would receive a value of 0.4, and the participant’s conversation partner’s contribution would be 0.6. If, however, the conversational dyad dis-entrained, i.e., became more dissimilar by 10 Hz from each other by the end of the conversation, then the participant who contributed 4 Hz to this change would have an entrainment contribution value of −0.4. On average, participants with ASD contributed 71% less to the convergence in mean f0 during the conversation compared to their neurotypical peers. Figure 3A shows the group differences in entrainment contribution and particularly illustrates that the ASD participants contributed more to dis-entrainment than to the convergence in mean f0. The second significant relationship was found between mean f0 entrainment contribution and language ability as measured by the Core Language subtest of the CELF (*b* = −0.03, *SE* = 0.01, *t* = −2.59, *p* = 0.02). Study participant with better language ability contributed less to the convergence in mean f0 compared to participants with lower language ability scores. This was true for all participants regardless of whether they carried an ASD diagnosis or not. This relationship between language ability and mean f0 entrainment contribution is illustrated in Figure 3B.

No significant relationships between mean f0 entrainment contribution and either the age, gender, or non-verbal IQ of the participant were found. There was also no relationship between mean f0 entrainment contribution and conversation partner. The linear regression model with mean f0 entrainment contribution as main dependent variable and “Group” and “CELF” as predictor variables explains 45% (Adjusted *R*^2^ = 0.446) of the variance in mean f0 entrainment contribution found in our data set. Group differences alone describe 27% (Adjusted *R*^2^ = 0.269) of the variance in mean f0 entrainment contribution.

#### Adjusted Mean f0 Entrainment Contribution

**FIGURE 4:**
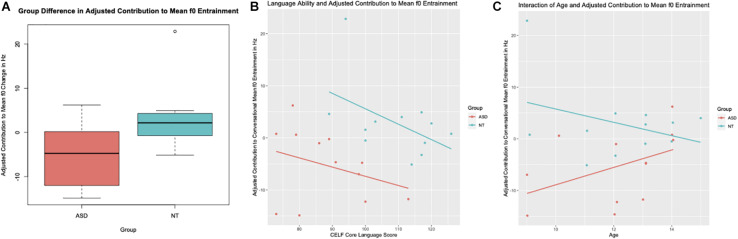
**(A)** Group Differences between children and teens with Autism Spectrum Disorder (ASD) and their neurotypical peers (NT) in the contribution to mean f0 entrapment adjusted for the overall amount of entrainment of the conversation. **(B)** Relationship between language ability as measured by the Core Language sub-test of the CELF and the adjusted mean f0 entrainment contribution. **(C)** The group interaction between age in years and adjusted contribution to mean f0 entrainment.

As explained earlier, the adjusted mean f0 entrainment contribution variable combines the measures of mean f0 entrainment for the conversation and the individual participant’s contribution to this entrainment. It hence combines aspects of the conversation as a whole with aspects of the individual speaker’s contribution. The relationship between adjusted entrainment contribution and the group variable was again significant (*b* = 51.68, *SE* = 17.45, *t* = 2.96, *p* = 0.008). While participants in the ASD group moved on average 5.3 Hz away from their conversation partners during the conversation, neurotypical participants moved on average 3 Hz closer to their conversation partners’ f0. Figure 4A shows the group difference in adjusted mean f0 entrainment contribution. A significant relationship was also found between adjusted entrainment contribution and language ability (*b* = −0.25, *SE* = 0.11, *t* = −2.26, *p* = 0.035). Similar to the unadjusted entrainment contribution variable, participants in both groups with higher CELF Core Language scores entrained less than participants with lower language scores. The relationship between language ability and adjusted mean f0 entrainment contribution is shown in Figure 4B. Furthermore, a significant interaction between “Group” and “Age” was fund (*b* = −3.25, *SE* = 1.39, *t* = −2.26, *p* = 0.035). Similar to the interaction of these variables seen in overall mean f0 entrainment over the course of the conversation; older participants with ASD showed more convergence in mean f0 compared to younger ASD participants—while older neurotypical participants entrained less compared to younger neurotypical participants (see Figure 4C).

The linear regression, which modeled adjusted mean f0 entrainment contribution as main dependent variable and “Group,” “CELF,” and the interaction of “Age”^∗^ “Group” as predictor variables, explains 52% (Adjusted *R*^2^ = 0.520) of the variance in adjusted mean f0 entrainment contribution.

### Fundamental Frequency Range Entrainment

Looking at the percentage of conversations in each group that showed entrainment in fundamental frequency range over the course of the conversation, we find that out of the 12 conversations per group, four (33%) show a convergence in f0 range in either group. In order to investigate the relationship between (1) f0 range entrainment over the conversation, (2) contribution to the f0 range entrainment of each participant, and (3) the adjusted f0 range entrainment contribution further, we constructed a series of linear regression models akin to those used in the statistical analysis of mean f0 entrainment. No statistically significant relationship between either of the three dependent variables (f0 range entrainment over the conversation, f0 range entrainment contribution, or adjusted f0 range entrainment contribution) and the predictor variables “Group,” “Age,” “Gender,” “Non-verbal IQ,” and “CELF” at an alpha level < 0.05 were found. Therefore, we conclude that no group differences in f0 range entrainment exist in our data set.

## Discussion

The fact that many individuals with ASD show differences in speech prosody has been well established. The present study presents results that tie prosodic differences in those with ASD directly to the interpersonal aspect of conversational prosody. Prosodic entrainment in mean f0 and f0 range in goal-directed conversations was investigated in children and teens with and without an ASD diagnosis. Our results suggest that although a small number of our participants with ASD did entrain to their conversation partner in mean f0, as a group, study participants with ASD differed significantly from their neurotypical peers in mean f0 entrainment over the course of a conversation. In contrast, although youth with ASD did differ significantly in f0 range from the control group, we observed no group differences in f0 range entrainment at the conversational level. This is surprising given that differences in f0 range production in those with ASD is one of the prosodic traits that have been found in numerous studies (Diehl et al., 2009; Green and Tobin, 2009; Sharda et al., 2010; DePape et al., 2012; Nadig and Shaw, 2012). On the other hand, these differences in f0 range production of those with ASD do not seem to contribute to the perceived oddness of the prosody of those with ASD (Nadig and Shaw, 2012; Patel et al., 2020). Perhaps the absence of a difference in f0 range entrainment is further evidence that differences in f0 range in those with ASD, albeit common, do not constitute a barrier to successful communication and social integration. However, it is also possible that the lack f0 range entrainment is due to the anatomical and physiological changes that take place during the age range of our participants (9–15 years). This age range coincides with a temporary reduction in f0 range or variability. Acoustic measures of children’s f0 and f0 variability compared to acoustic measures of adult speakers have shown that for boys, f0 variation significantly decreases between 12 and 15 years of age and for girls between 10 and 14 years (Lee et al., 1999). Yet another possibility is that entrainment in f0 range—since it is to a large extend reflected in the intonation contours used by speakers—is more amenable to f0 entrainment expressed as synchrony rather than convergence, since similar intonational contours may be used by conversation partners in adjacent turns to express entrainment (Gravano et al., 2014). As pointed out earlier, the current study did not assess synchronous entrainment behavior at the level of the conversational turn, and, therefore, this remains a topic for future investigation.

Given the positive correlation between f0 entrainment and the success of conversations as well as personal and interpersonal attributes of the conversation partners (Lubold et al., 2016; Michalsky and Schoormann, 2017; Michalsky et al., 2018; Niebuhr and Michalsky, 2019), the finding that children and teens with ASD show less mean f0 entrainment compared to their neurotypical peers is significant. Especially, seen that earlier studies on lexical and speech rate entrainment did not find differences between participants with and without ASD (Nadig et al., 2015; Branigan et al., 2016; Hopkins et al., 2017; Wynn et al., 2018). Considering that deficits in social communication in those with ASD are considered a core-deficit in this population, measures of f0 entrainment behavior have the potential to serve as a biomarker for assessing both the presence and the severity of the social communication deficit in this population, and possibly in others with social communication deficits, directly. Therefore, the results presented here show that further investigation of prosodic entrainment in those with ASD is warranted. In particular, the relationship between prosodic entrainment and measures of social communication performance will need to be established next in order to show the validity of f0 entrainment measures as indicators of social communication deficits.

An innovation of the current study is that—unlike earlier studies of conversational speech in those with ASD (Green and Tobin, 2009; Sharda et al., 2010; Nadig and Shaw, 2012)—this study investigates conversational prosody over large segments of conversational speech and in relation to the conversational behavior of each study participants’ conversation partner. One of the notable findings in this respect is the interaction of age and mean f0 entrainment (see section “Mean f0 Conversational Entrainment”). Conversations between participants with ASD and their adult research assistant show more entrainment in mean f0 if the participant with ASD was older compared to younger participants with ASD. However, we find the inverse pattern in the conversations with our neurotypical controls (see Figure 2), namely conversations with older participants tend to exhibit less entrainment—a pattern also described by Nilsenova et al. (2009). However, we clearly see that the group difference in terms of the interaction between age and mean f0 disappears when we take only the individual contribution of each participant into account (see section “Mean f0 Entrainment Contribution”). This suggests that what at first looks like an atypical developmental trajectory in the entrainment behavior of youth with ASD is actually driven by the prosodic entrainment behavior of their conversation partners. In other words, when conversation partners interacted with neurotypical participants, they did not show an increase in their mean f0 entrainment in response to a reduced entrainment in the older participants. In contrast, conversation partners did show compensation for reduced entrainment in older participants with ASD, exhibiting an increase in mean f0 entrainment in these conversations. These results open up a different venue of investigation, namely that of how those who interact with individuals with ASD adjust their conversational style and what specific aspects in the speech (or other yet to be identified characteristics) of those with ASD prompts such adjustment on the part of conversation partners.

kOn the other hand, we observe that there is only a marginal relationship between language ability of the child/teen participants and mean f0 entrainment when considering the overall mean f0 entrainment of the conversational dyads. However, when considering each individual participant’s contribution to mean f0 entrainment (see section “Mean f0 Entrainment Contribution”), it becomes clear that those children and teens with higher scores on standardized language tests entrain less to their conversation partners, regardless of ASD diagnosis. This suggests that the conversation partners of those study participants with higher language ability responded to reduced entrainment in those participants by entraining more in mean f0. Interestingly, this happened regardless of whether the participant was diagnosed with ASD or not, possibly indicating that language ability does not influence conversation partners in a way that elicits a differential response to those with ASD.

The results reported here show potential for prosodic entrainment, in particular mean f0 entrainment, as a fruitful area of research for the exploration of prosody differences in the speech of those with ASD. Several new questions arise from the findings presented here; first and foremost, more research is needed to investigate whether prosodic entrainment is indicative of the degree of social impairment in those with ASD. Furthermore, the perceptual aspects of prosodic entrainment in those with ASD need to be investigated. In particular, it will be important to investigate whether differences in entrainment are perceived by those with ASD in a comparable way to neurotypical peers. Similarly, it is important to explore how neurotypical peers interpret the differences in prosodic entrainment behaviors in those with ASD and whether these differences constitute a barrier to successful social communication and integration of those with ASD. An important next step to accomplish these research goals is to broaden the current approach and investigate the ongoing process of the coordination of prosodic features throughout the conversation, including entrainment behavior at the level of the conversational turn. This will help to gain more insight into whether there are differences in the locus of entrainment between those with and without ASD.

The current study is limited in several ways. More research is needed to confirm the findings reported here with a larger sample size. Furthermore, we are drawing conclusions about the entrainment behaviors of our participants based on a single conversation. While it is inherently difficult to investigate conversational behavior in those with ASD due to the fact that many individuals with ASD find engaging in conversations challenging, it would be important to investigate entrainment behavior across multiple conversations with different conversation partners to obtain a more complete picture of entrainment behaviors in those with ASD.

## Conclusion

In conclusion, the current study has presented the first evidence that children and adolescents with ASD differ from their neurotypical peers in conversational mean f0 entrainment such that youth with ASD tend to dis-entrain from their conversation partners over the course of a conversation while neurotypical peers show convergence in mean f0. Differences in the interaction of mean f0 entrainment and age between participants with and without ASD were shown to be due to the entrainment behavior of the conversation partners rather than to the entrainment behavior of those with ASD. It was generally the case that children and teens with better language skills entrained less, regardless of ASD diagnosis. Future research will have to show whether and to what extend these differences in prosodic entrainment correlate with perceptions of social communicative competence in those with and without ASD.

## Data Availability Statement

The data supporting the conclusions of this article will be made available by the authors, without undue reservation, to any qualified researcher.

## Ethics Statement

The studies involving human participants were reviewed and approved by the New Mexico State University Institutional Review Board. Written informed consent to participate in this study was provided by the participants’ legal guardian/next of kin.

## Author Contributions

HL-L designed study, coordinated and oversaw data collection, administered standardized tests, conducted statistical analysis, and prepared the manuscript. ST and SS conducted the acoustic analysis, developed entrainment measures, and contributed to the description of these analyses in the manuscript. All authors proofread manuscript.

## Conflict of Interest

The authors declare that the research was conducted in the absence of any commercial or financial relationships that could be construed as a potential conflict of interest.
